# Higher Peritoneal Protein Clearance as a Risk Factor for Cardiovascular Disease in Peritoneal Dialysis Patient

**DOI:** 10.1371/journal.pone.0056223

**Published:** 2013-02-13

**Authors:** Tae Ik Chang, Ea Wha Kang, Yong Kyu Lee, Sug Kyun Shin

**Affiliations:** Department of Internal Medicine, NHIC Medical Center, Ilsan Hospital, Goyangshi, Gyeonggi–do, Republic of Korea; University of Louisville, United States of America

## Abstract

**Background and Aims:**

Although a number of studies have been published on peritoneal protein clearance (PrCl) and its association with patient outcomes, the results have been inconsistent. Therefore, the intent of this study was to evaluate the impact of PrCl on cardiovascular disease (CVD) and mortality in peritoneal dialysis (PD) patients.

**Methods:**

This prospective observational study included a total of 540 incident patients who started PD at NHIC Ilsan Hospital, Korea from January 2000 to December 2009. Two different types of analyses such as intention-to-treat and as-treated were used.

**Results:**

Correlation analyses revealed that PrCl was positively correlated with diabetes, pulse pressure, C–reactive protein (CRP) level, dialysate/plasma creatinine ratio (D/P cr) at 4 h, and peritoneal Kt/V urea. PrCl was inversely correlated with serum albumin and triglyceride levels. On multivariate analysis, serum albumin, pulse pressure, D/P cr at 4 h, and peritoneal Kt/V urea were found to be independent determinants of PrCl. A total of 129 (23.9%) patients in intention-to-treat analysis and 117 (21.7%) patients in as-treated analysis developed new cardiovascular events. Time to occurrence of cardiovascular event was significantly longer in patients with a value of PrCl below the median (89.4 ml/day). In multivariate analysis, older age, presence of diabetes or previous CVD, and higher PrCl were independent predictors of cardiovascular events. Patients above the median value of PrCl had a significantly lower rate of survival than those below the median. However, a higher PrCl was not associated with increased mortality in multivariate Cox analysis.

**Conclusions:**

A higher PrCl is a risk for occurrence of cardiovascular event, but not mortality in PD patients. Large randomized clinical trials are warranted to confirm this finding.

## Introduction

Peritoneal dialysis (PD) is an established treatment modality in end-stage renal disease (ESRD) patients and approximately 150000 patients are being maintained on PD worldwide [1]. In Korea, PD was introduced in the early 1980s and has been performed in many centers so far. Currently, more than 7500 Korean patients are maintained on PD. Recently, there have been significant improvements in patient outcomes due to advances such as the optimization of the adequacy of dialysis, management of blood pressure and anemia, and the maintenance of biochemical parameters within the target range. However, their morbidities and mortality have been much higher than those of the general population. Specifically, cardiovascular disease (CVD) is the most important cause of hospitalization and death in this population. Therefore, the management of CVD is one of the primary goals in treating patients with ESRD on PD [2]. Although the pathogenesis of CVD in PD patients is complicated and not completely understood, non-traditional as well as traditional risk factors such as diabetes, hypertension, smoking, and dyslipidemia are all associated with the high prevalence of CVD in PD patients [3]. Chronic inflammation and malnutrition are risk factors specifically related to ESRD patients on chronic PD which play a pivotal role in the development of CVD in these patients [4].

Although waste products are cleared during dialysis treatment, nutrients are also lost into the dialysate. Patients on PD may lose approximately 9–12 g of total protein and 6–8 g of albumin daily through the peritoneal membrane [5], [6]. Variability in peritoneal protein loss both within and between patients is high, which can be explained by the dependency of protein transport on both the effective peritoneal surface area (the number of mainly small pores) and the intrinsic size-selective permeability (the diameter of the large pores) [7]. Protein loss during dialysis is usually compensated for by an increase in albumin synthesis in PD patients [8]. However, this process can be suppressed in cases of coexisting inflammation and malnutrition. In addition, the type of peritoneal membrane transport might influence the amount of protein loss. Protein loss is much greater in patients with a fast peritoneal solute transport rate than in those with a low solute transport rate [9]. Fast peritoneal solute transport rates have also been associated with hypoalbuminemia [10], inflammation [11], mortality, and technique failure [12]–[14] in some studies, but this is not always the case [15]–[17].

During the last decade, a number of studies have been published on peritoneal protein clearance (PrCl) and its association with outcomes in PD patients [18]–[25]. In some studies, PrCl has been shown to be associated with a higher prevalence of CVD and increased mortality in PD patients [18]–[22]. However, other studies have found no association between PrCl and an increased in cardiovascular events or mortality [23]–[25]. Therefore, the intent of this study was to investigate the impact of PrCl on mortality and the occurrence of cardiovascular events and to identify the determinants of PrCl in a large prospective cohort of incident patients who were on PD for at least six months.

## Methods

### Ethics statement

The study was carried out in accordance with the Declaration of Helsinki and approved by the Institutional Review Board of Ilsan Hospital Clinical Trial Center. We obtained informed written consent from all participants involved in our study.

### Patients and data collection

We considered all of 620 patients who started PD at NHIC Ilsan hospital, one of the largest PD centers in Korea, from January 2000 to December 2009. We then excluded patients that were younger than 18 years of age at initiation of PD, patients that had less than 6 months of follow-up, and patients that had been on hemodialysis or received a kidney transplant before PD. Patients that recovered kidney function or started PD for other reasons, such as acute renal failure or congestive heart failure, were also excluded from the analysis. Therefore, this prospective observational study included a total of 540 incident patients finally. All of the patients underwent a peritoneal equilibration test, measurement of dialysis adequacy, and a 24-h dialysate PrCl analysis within three months of PD initiation. Demographic and clinical data were collected at the beginning of PD. The data recorded included age, gender, body mass index (BMI) calculated as weight/(height)^2^, duration of PD, cause of ESRD, prevalence of diabetes and CVD, blood pressure, and patient outcomes. Laboratory data obtained at the time of dialysis adequacy measurement were considered baseline values and included blood urea nitrogen, serum creatinine, total cholesterol, serum triglyceride, serum albumin concentrations, serum C-reactive protein (CRP) levels, Kt/V urea, percentage of lean body mass (%LBM), normalized protein catabolic rate (nPCR), residual glomerular filtration rate (GFR), and 24-h residual urine volume. Residual GFR was calculated as the average urea and creatinine clearance from a 24-h urine collection [26].

Peritoneal dialysate protein losses were measured from the collection of 24-h peritoneal dialysate effluent by the pyrogallol red method. Serum and dialysate concentrations of urea, creatinine, glucose, and albumin were all measured by a colorimetric method (UniCel DXC 800; Beckman Coulter, Inc., California, USA). To calculate PrCl, the total 24-h dialysate protein loss was divided by a validated correction factor (serum albumin/0.4783) which has been used in other previous studies [22], [23]. PrCl was expressed as ml of plasma cleared per day.

### Statistical analysis

All values are expressed as mean ± standard deviation or percentages. Statistical analyses were performed using SPSS for Windows version 13.0 (SPSS, Inc., Chicago, IL, USA). Data were analyzed using Student's *t*–test and the chi-square test. The relationship between PrCl and continuous variables was examined by Pearson's correlation coefficient, and categorical variables were examined using Spearman's *R* test. Multiple linear regression analysis was performed to identify the determinants of PrCl using two different models with or without serum albumin because of the fact that serum albumin levels inevitably interfered in the estimation of PrCl. Primary outcomes were death and newly developed cardiovascular events, which were defined as coronary artery disease (angioplasty, coronary artery bypass graft, myocardial infarction, or angina), cerebrovascular disease (transient ischemic attack, stroke, or carotid endarterectomy), and peripheral artery disease (revascularization or amputation). We used both intention-to-treat and as-treated analyses. In the former, we followed patients from the date of dialysis initiation to the earlier of death or December 31, 2011. In the latter, we followed patients from the date of dialysis initiation to the earlier of death, change to hemodialysis (88 patients), renal transplantation (36 patients), loss to follow-up (29 patients), or December 31, 2011. To determine risk factors for cardiovascular events and mortality, multivariate Cox regression was performed, and all significant covariates from the univariate analysis were included. Survival was also examined by comparing patients above and below the median value of PrCl using the Kaplan-Meier method and the log-rank test. A *p*–value of less than 0.05 was considered statistically significant.

## Results

### Demographic and clinical data

Table 1 details baseline characteristics of the 540 incident PD patients. The mean age of the patients was 59 years (range 20 to 88 years), 53.7.1% were males, and patients were on PD for a mean of 50 months (range 6 to 142 months). The prevalence of diabetes and CVD was 53.3% and 28%, respectively. Total 24-h peritoneal protein loss was 6.7±4.4 g/day and the mean PrCl was 107.3±82.8 ml/day (median 89.4 ml/day, range 22.7 to 617.7 ml/day).

**Table 1:** Baseline characteristics of the study subjects (n = 540).

Age (years)	59.1	±	14.0
Gender (Male)	290		(53.7)
Body mass index (kg/m^2^)	22.8	±	6.2
Pulse pressure (mmHg)	56.6	±	17.5
Follow-up duration (months)	50.2	±	33.9
Underlying renal disease			
Diabetes mellitus	258		(47.8)
Hypertension	155		(28.7)
Chronic glomerulonephritis	76		(14.1)
Polycystic kidney disease	9		(1.7)
Others	15		(2.8)
Unknown	27		(5.0)
Presence of diabetes mellitus	288		(53.3)
Presence of prior cardiovascular disease	151		(28.0)
Laboratory findings			
Serum albumin (g/dL)	3.2	±	1.6
Total cholesterol (mg/dL)	183.6	±	48.6
Triglyceride (mg/dL)	150.6	±	91.4
CRP (mg/dL)	0.7	±	2.6
Peritoneal transport (D/P cr at 4 h)	0.68		0.18
Dialysis adequacy			
Peritoneal Kt/V urea	1.5	±	0.6
24-h dialysate protein loss (g)	6.7	±	4.4
24-h PrCl (mL)	107.3	±	82.8
24-h residual urine volume (mL)	1083.9	±	719.1
Residual GFR (mL/min/1.73 m^2^)	6.1	±	5.2
nPCR (g/Kg/day)	1.0	±	0.3
Lean body mass (% body weight)	66.0	±	28.5

Data are expressed as mean ± standard deviation or number of patients (percent).

CRP, C-reactive protein; D/P cr, dialysate/plasma creatinine ratio; PrCl, peritoneal protein clearance; GFR, residual glomerular filtration rate; nPCR, normalized protein catabolic rate.

Gender, prevalence of CVD, and residual renal function (RRF) were not different between the groups when patients were divided into two groups (low and high) based on the median levels of baseline PrCl. Age (60.4±13.5 *versus* 57.8±14.4 years, *P* = 0.026), prevalence of diabetes (58.8 *versus* 47.8%, *P* = 0.01), serum log_10_CRP level (−0.79±0.79 *versus* −0.95±0.71 mg/dL, *P* = 0.022), D/P cr at 4 h (0.72±0.18 *versus* 0.64±0.16, *P*<0.001), and peritoneal Kt/V (1.5±0.6 *versus* 1.4±0.5, *P* = 0.044) were significantly higher, while serum triglyceride (137.3±85.5 *versus* 163.9±95.2 mg/dL, *P* = 0.001) and albumin levels (2.9±0.5 *versus* 3.4±0.5 g/dL, *P*<0.001), and nPCR (0.95±0.24 *versus* 1.01±0.27 g/Kg/day, *P* = 0.004) were significantly lower in the high PrCl group compared to the low PrCl group.

### Factors associated with peritoneal protein clearance

Correlation analyses were performed to identify factors associated with PrCl. PrCl was positively correlated with the presence of diabetes (γ = 0.1, *P* = 0.005), pulse pressure (γ = 0.11, *P* = 0.016), log_10_CRP level (γ = 0.096, *P* = 0.033), D/Pcr at 4 h (γ = 0.13, *P* = 0.004), and peritoneal Kt/V urea (γ = 0.22, *P*<0.001), whereas it was inversely correlated with serum albumin concentration (γ = −0.37, *P*<0.001), and triglyceride level (γ = −0.09, *P* = 0.038). In contrast, there was no correlation between age, gender, BMI, presence of preexisting CVD, RRF, nPCR or %LBM and PrCl. On multivariate linear regression analysis, serum albumin, D/Pcr, peritoneal Kt/V urea, and pulse pressure were independently associated with PrCl (Table 2; Model 1). In model 2, in which albumin was excluded, D/Pcr, peritoneal Kt/V urea, and pulse pressure remained in the model with the additional inclusion of diabetes and increased serum CRP levels (Table 2; Model 2).

**Table 2:** Multivariate associations between peritoneal protein clearance and patient characteristics^a^.

	Model 1		Model 2	
	β	*P*-value	β	*P*-value
Serum albumin (g/dL)	−0.310	<0.001	-	-
D/P cr at 4 h	0.134	0.002	0.194	<0.001
Peritoneal Kt/V urea	0.311	<0.001	0.314	<0.001
Pulse pressure (mmHg)	0.085	0.046	0.094	0.037
Presence of diabetes	0.084	0.062	0.169	<0.001
Log_10_CRP (mg/dL)	0.070	0.106	0.111	0.014
Triglyceride (mg/dL)	0.001	0.998	−0.048	0.278

a Model 1 includes albumin; model 2 excludes serum albumin.

CRP, C-reactive protein; D/P cr, dialysate/plasma creatinine ratio.

### Predictors of cardiovascular events

**Figure 1:**
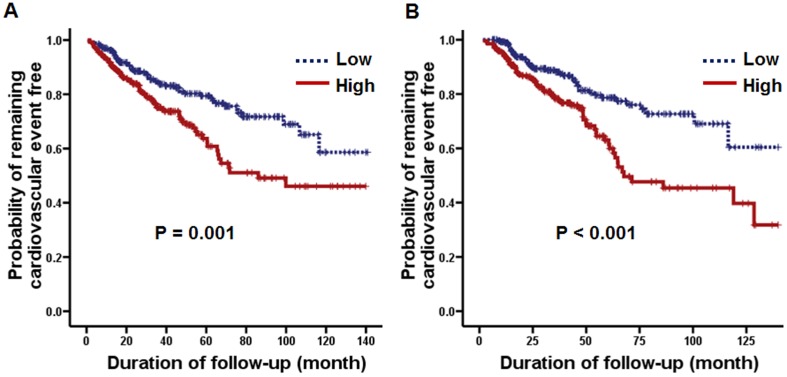
Kaplan-Meier plots of the probability of remaining cardiovascular event-free based on the median level (89.4 ml/day) of baseline peritoneal protein. (A) Intention-to-treat analysis. (B) As-treated analysis.

In intention-to-treat analysis, 129 (23.9%) patients developed new cardiovascular events, which included coronary artery disease (73 patients, 56.6%), cerebrovascular disease (44 patients, 34.1%), and peripheral artery disease (12 patients, 9.3%). Additionally, in as-treated analysis, 117 (21.7%) patients developed new cardiovascular events. In both intention-to-treat and as-treated analyses, time to occurrence of cardiovascular event was significantly longer in patients with a value of PrCl below the median (Figure 1). In the univariate Cox proportional hazards model, age, presence of diabetes or preexisting CVD, serum albumin concentration, %LBM, and PrCl were significantly associated with cardiovascular events, whereas CRP level, peritoneal Kt/V urea, nPCR, and RRF were not associated with cardiovascular events. In multivariate analysis, older age, presence of diabetes or previous CVD, and higher PrCl were independent predictors of cardiovascular events (Table 3). Furthermore, we performed additional analysis using measured total peritoneal protein loss, not only the calculated clearance. Even when we considered absolute peritoneal protein amount with or without correction to body surface area, an association with increased cardiovascular events persisted (details not shown).

**Table 3:** Multivariate Cox regression analysis for cardiovascular events.

	Intention to Treat			As Treated		
	HR	(95% CI)	*P*-value	HR	(95% CI)	*P*-value
Age (years)	1.02	(1.00–1.03)	0.022	1.02	(1.01–1.04)	0.012
Presence of diabetes	1.96	(1.33–2.86)	0.001	1.95	(1.29–2.94)	0.002
Presence of prior CVD	1.67	(1.14–2.44)	0.008	1.72	(1.15–2.57)	0.009
Serum albumin (g/dL)	0.85	(0.59–1.22)	0.374	0.74	(0.51–1.08)	0.743
Lean body mass (% body weight)	0.99	(0.98–1.01)	0.388	0.99	(0.98–1.01)	0.367
PrCl (per 10 ml/day increase)	1.02	(1.00–1.03)	0.041	1.02	(1.00–1.04)	0.025

HR, hazard ratio; CI, confidence interval; CVD, cardiovascular disease; nPCR, normalized protein catabolic rate; PrCl, peritoneal protein clearance.

### Predictors of mortality

**Figure 2:**
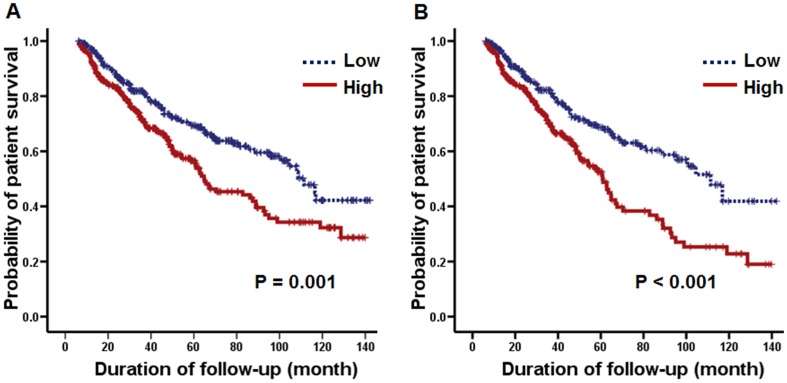
Kaplan-Meier plots for patient survival based on the median level (89.4 ml/day) of baseline peritoneal protein clearance. (A) Intention-to-treat analysis. (B) As-treated analysis.

In intention-to-treat analysis, 203 (37.6%) deaths were recorded, and the median survival period was 42 months (range 6 to 142.2 months). CVD (37.9%) was the most common cause of death in this study, followed by infection (32.0%), unknown cause (22.7%), and others (7.4%). Additionally, in as-treated analysis, 187 (21.7%) deaths were recorded. In both intension-to-treat and as-treated analyses, patients above the median value of PrCl had a significantly lower rate of survival than those below the median (Figure 2). In the univariate Cox proportional hazards model, age, presence of diabetes or CVD, serum albumin concentration, Log_10_CRP level, peritoneal Kt/V urea, nPCR, %LBM, RRF, and PrCl were significantly associated with mortality. However, gender, BMI, pulse pressure, serum triglyceride level, and small solute transport status (D/Pcr at 4 h) were not associated with mortality. In multivariate analysis, older age, presence of diabetes or prior CVD, and hypoalbuminemia were independent predictors of mortality, whereas RRF was also identified as a significant risk factor for mortality in as-treated analysis (Table 4).

**Table 4:** Multivariate Cox regression analysis for mortality.

	Intention to Treat			As Treated		
	HR	(95% CI)	*P*-value	HR	(95% CI)	*P*-value
Age (years)	1.06	(1.04–1.08)	<0.001	1.07	(1.05–1.08)	<0.001
Presence of diabetes	1.56	(1.11–2.19)	0.010	1.58	(1.10–2.25)	0.012
Presence of prior CVD	1.61	(1.17–2.22)	0.004	1.70	(1.21–2.37)	0.002
Serum albumin (g/dL)	0.50	(0.37–0.68)	<0.001	0.48	(0.35–0.66)	<0.001
Log_10_CRP (mg/dL)	1.20	(0.97–1.48)	0.102	1.19	(0.96–1.47)	0.112
Peritoneal Kt/V urea	1.29	(0.95–1.77)	0.108	1.12	(0.81–1.57)	0.491
nPCR (g/Kg/day)	0.80	(0.38–1.67)	0.550	0.91	(0.43–1.95)	0.813
Lean body mass (% body weight)	0.99	(0.98–1.01)	0.317	1.00	(0.98–1.01)	0.632
Residual GFR (mL/min/1.73 m^2^)	0.96	(0.92–1.01)	0.106	0.93	(0.88–0.98)	0.005
PrCl (per 10 ml/day increase)	1.00	(1.00–1.00)	0.425	1.01	(0.99–1.03)	0.260

HR, hazard ratio; CI, confidence interval; CVD, cardiovascular disease; CRP, C-reactive protein; nPCR, normalized protein catabolic rate; GFR, residual glomerular filtration rate; PrCl, peritoneal protein clearance.

## Discussion

This study, which included a total of 540 incident PD patients, is the largest one published to date on peritoneal transport of total protein and its association with patient outcomes in terms of cardiovascular events and mortality. The results demonstrated that a higher PrCl is a risk factor for occurrence of cardiovascular event, but not mortality in patients treated with PD.

Peritoneal protein clearance in PD patients reflects protein leakage across the large pores, which is equivalent to large-pore flow (JvL). Although a number of studies have been published on PrCl and its association with patient outcomes, the results have been inconsistent, and no consensus has been reached on the relationship and significance of PrCl in the development of subsequent cardiovascular events and death in PD patients [18]–[25]. Szeto *et al*. [18] showed that patients starting PD with active CVD had higher protein and albumin levels in the peritoneal effluent, and cardiovascular events were more frequent in patients with greater peritoneal albumin losses. The authors postulated that peritoneal protein losses or PrCl could be an independent cardiovascular risk factor, and is likely associated with high mortality in PD patients. In addition, Heaf *et al*. [20] found that JvL is related to hypoalbuminemia and mortality after PD initiation, and Van Biesen *et al*. [21] found that a higher JvL, when corrected for membrane area parameter (A0/Δx), is a marker of inflammation and is related to decreased patient survival. Another recent study by Peal *et al*. [22] revealed that increased PrCl at the start of PD is a predictor of death independent of baseline small solute transport status, serum albumin, age, and comorbidity. All of the above studies support the hypothesis that protein leakage across the membrane may be a manifestation of local or systemic inflammation as likely as microalbuminuria, which is another manifestation of capillary protein leakage, and is recognized as an endothelial dysfunction marker [27]. In contrast to the aforementioned studies, no association between PrCl and an increase in cardiovascular events or mortality was found in other studies [23]–[25]. Sanchez-Villanueva *et al.* [23] showed that PrCl is significantly and independently related to the presence of peripheral artery disease, but failed to demonstrate the association of PrCl with mortality or subsequent cardiovascular events. In addition, a recent study by Balafa *et al.* [25], which was the largest published to date, reported that PrCl was not an independent predictor of mortality. The authors postulated that the association of increased albumin leakage with inflammation is only consequence of the availability of an increased number of large pores and is not related to endothelial dysfunction or CVD. This discrepancy between findings is likely due to differences in study design, the number of enrolled patients, patient characteristics, timing and methods of measuring PrCl, and variables included in the multivariate analysis.

This study of long term (up to 142 month) clinical outcomes of a large cohort of patients treated with PD provides strong evidence of that a higher PrCl is a risk factor for occurrence of cardiovascular event in these patients. These findings were observed consistently in both intention-to-treat and as-treated analyses. Even when we considered absolute protein losses corrected to body surface area, an association with reduced survival of remaining cardiovascular event-free persisted. On the other hand, our study on mortality does not significantly demonstrate differences according to initial PrCl. We, however, reconfirmed that older age, presence of diabetes or previous CVD, hypoalbuminemia, and lower RRF, all of which are well-known risk factors, are independent predictors of mortality in PD patients. The underlying mechanisms for these poor outcomes in patients with higher PrCl are unclear. One possible explanation could be the link between CVD and endothelial dysfunction as evidenced by microalbuminuria [27]. Here, we found that higher peritoneal protein losses or PrCl were significantly associated with lower serum albumin concentrations, which might reflect a high inflammatory and poor nutritional status [28], [29] and a higher pulse pressure, which might reflect arterial stiffness and systemic vascular damage [30]. In addition, higher serum CRP values, which were available in 494 patients in this study, was also associated with higher PrCl, which although did not reach statistical significance on multivariate analysis. In line with our results, a recent cross-sectional study of prevalent PD patients from Korea [31] showed that PrCl exhibited a positive correlation with both heart-to-femoral pulse wave velocity implicating arterial stiffness and peripheral vascular calcium scores, which is representative of vascular calcification. Along with systemic inflammation, local inflammation may also contribute to CVD for those on PD. With high dialysate leukocyte count and IL-6 concentration, the peritoneal protein or albumin leakage has been considered to be one of these intra-peritoneal inflammation-related factors in PD patients [18], [32]. Taken together, we surmised that PrCl could represent systemic or local vascular damage and endothelial dysfunction in PD patients. Another possible factor to note is that patients with a higher PrCl might have poor nutritional status. In our results, BMI, nPCR, and serum triglyceride concentration were significantly lower in patients above the median PrCl value irrespective of the presence of hypoalbuminemia. Other nutritional markers such as total cholesterol and %LBM were also lower in patients with a higher PrCl, although the differences were not statistically significant. Based on these findings, it might be possible to postulate that these poor outcomes of patients with higher PrCl are mediated, at least partially, by the poor preserved nutritional status seen with protein–energy wasting, which has been shown to be an important predictor of morbidity and mortality in PD patients [33]–[35]. However, other mechanisms that remain to be explored may also play a role.

The strengths of this study are the large number of patients, the long duration of follow-up, and the adjustment for multiple potentially confounding covariates such as serum CRP. Potential limitations are the single-center design of the study and the fact that data from a full panel of peritoneal inflammation markers were not available. The lack of urinary protein excretion measurements limited the testing of possible relationships between urinary and peritoneal protein losses as a result of the common pathway of endothelial dysfunction.

## Conclusions

This study showed that baseline PrCl is a risk factor for occurrence of cardiovascular event, but not mortality in patients on PD. These results suggest that protein leakage across the membrane may be a manifestation of a systemic or local inflammation and poor nutritional status, serving as a surrogate marker for the increased morbidity seen with malnutrition, inflammation, and atherosclerosis syndrome. A large, prospective study is required to further elucidate the association between PrCl and inflammatory or nutritional status in PD patients. Further investigations focusing on the description of changes in PrCl with time on treatment and the associated prognostic significance are also warranted.
